# Left ventriculum diastolic dysfunction in pediatric septic shock

**DOI:** 10.1186/cc10800

**Published:** 2012-03-20

**Authors:** M Georgiyants, V Korsunov

**Affiliations:** 1 Kharkov Medical Academy Post-Graduate Education, Kharkov, Ukraine

## Introduction

One of the causes of septic mortality is a low cardiac output secondary to preload failure. Same patients demonstrate preload failure after aggressive volume replacement [1].

## Methods

Ultrasound impulse-wave Doppler evaluation of transmitral flow: VmaxE, VmaxA, ejection time E,A; DT E wave, IVRT of LV. Ultrasound evaluation of end-diastolic and end-systolic LV volume, stroke volume (LVEDV, LVESV, SV) on Teichholz L. EDLVP = 1.06 + 15.15 × VTI peakA/VTI peakE. Coronary perfusion pressure (CPP) = EDLVP - diastolic BP. We evaluate these parameters in 34 patients (age 28.1 ± 8.0 months) with septic shock (SS) diagnosed according to Consensus 2002. Control (C) - 44 healthy children (age 40.7 ± 8.5 months). Statistical analyses with *t *criteria.

## Results

The increase of VmaxA and decrease of VmaxE in patients of SS are demonstrated. IVRT and DT are less than in control group. We evaluated a decrease in E/A proportion. EDLVP in patients was more, and CPP lower, than in controls. See Table 1.

**Table 1:** Diastolic function in pediatric septic shock

Value	SS	C	*P *value
VmaxA	81	65	**<**0.05
VmaxE	99	108	>0.05
ETA	80	102	0.01
ETE	102	149	0.001
DTE	51	93	0.001
IVRT	48	87	0.01
E/A	1.3	1.7	0.01
EDLVP	23	9.8	<0.001
CPP	22	50	0.001
EDLVV	46	65	<0.001
SV	14	26	0.001

## Conclusion

Pediatric SS accompanied with LV diastolic dysfunction, which decreases the effectiveness of volume restoration therapy, reduces preload and cardiac output.
